# Long non-coding RNA KCNQ1 overlapping transcript 1 promotes the progression of esophageal squamous cell carcinoma by adsorbing microRNA-133b

**DOI:** 10.6061/clinics/2021/e2175

**Published:** 2021-04-16

**Authors:** Haitao Xu, Jing Miao, Shuai Liu, Hongjian Liu, Lianguo Zhang, Qingguang Zhang

**Affiliations:** I Department of Thoracic Surgery, Binzhou Medical University Hospital, Binzhou, Shandong 256603, China.; II Department of Pediatrics, Binzhou People’s Hospital, Binzhou, Shandong 256603, China.

**Keywords:** KCNQ1OT1, miR-133b, *EGFR*, PI3K/AKT, ESCC

## Abstract

**OBJECTIVE:**

The long non-coding RNA (lncRNA) KCNQ1 overlapping transcript 1 (KCNQ1OT1) exerts vital regulatory functions in diverse tumors. However, the biological function of KCNQ1OT1 in esophageal squamous cell carcinoma (ESCC) remains unclear.

**METHODS:**

KCNQ1OT1 expression was detected in ESCC tissues using quantitative real-time polymerase chain reaction (qRT-PCR). Cell proliferation, apoptosis, migration, and invasion were detected by the CCK-8 assay, EdU assay, flow cytometry analysis, and Transwell experiments, respectively. Bioinformatics analysis, luciferase reporter experiments, and RNA immunoprecipitation assays were used to predict and validate the regulatory relationships between KCNQ1OT1, microRNA-133b (miR-133b) and epidermal growth factor receptor (EGFR).

**RESULTS:**

KCNQ1OT1 expression was remarkably upregulated in ESCC tissues and cell lines. Overexpression of KCNQ1OT1 markedly promoted ESCC cell proliferation, migration, and invasion and enhanced the expression of N-cadherin, *MMP-2*, and *MMP-9*, but inhibited apoptosis and E-cadherin expression in ESCC cell lines; KCNQ1OT1 knockdown exerted the opposite effects. KCNQ1OT1 could directly bind to miR-133b and suppress its expression, and miR-133b reversed the effects of KCNQ1OT1 overexpression in ESCC cells. MiR-133b reduced the expression of epidermal growth factor receptor (*EGFR*); further, KCNQ1OT1 activated the phosphatidylinositol 3-kinase/AKT serine/threonine kinase 1 (PI3K/AKT) signaling pathway by repressing miR-133b repression and indirectly upregulating *EGFR*. KCNQ1OT1 expression was positively correlated with *EGFR* mRNA expression and negatively correlated with miR-133b expression.

**CONCLUSION:**

KCNQ1OT1 facilitates ESCC progression by sponging miR-133b and activating the EGFR/PI3K/AKT pathway.

## INTRODUCTION

Esophageal cancer (EC) is one of the most common gastrointestinal malignancies worldwide (1). Esophageal squamous cell carcinoma (ESCC) is the main pathological type of EC and is one of the leading causes of cancer-associated deaths worldwide (2). The 5-year survival rate of ESCC patients is only about 19%, and the median survival time of patients with advanced ESCC is less than 1 year (3-5). Therefore, it is necessary to study the molecular mechanism underlying the pathogenesis of ESCC and identify therapeutic targets for improving patient prognosis.

Long non-coding RNAs (lncRNAs) are evolutionarily conserved RNA molecules longer than 200 bases that have no or limited protein-coding functions (6,7). Recent studies have shown that lncRNAs play an important role in the tumorigenesis and development of various cancers (8,9). LncRNAs are frequently dysregulated in EC tissues and participate in cancer progression (10,11). For example, both linc-UBC1 and lncRNA-ATB are upregulated in ESCC, and their high expression levels indicate an adverse prognosis in patients with ESCC. Functionally, linc-UBC1 and lncRNA-ATB facilitate the growth and metastasis of ESCC (12,13). KCNQ1 overlapping transcript 1 (KCNQ1OT1) is implicated in the pathogenesis of diverse carcinomas including non-small cell lung cancer (NSCLC), gastric cancer, and ovarian cancer (14- 17). In ESCC, KCNQ1OT1 expression is upregulated; this is strongly associated with a shorter survival time in patients (18). Nevertheless, the biological function of KCNQ1OT1 in ESCC and the mechanisms underlying these functions remain unclear.

LncRNAs can participate in EC development by interacting with microRNAs (miRNAs) as competing endogenous RNAs (ceRNAs) (19). MiRNAs are small non-coding RNA (ncRNA) molecules that perform vital functions in various biological processes (20). Reportedly, miR-133b impedes the phosphatidylinositol 3-kinase/AKT serine/threonine kinase 1(PI3K/AKT) signaling pathway by targeting the epidermal growth factor receptor (EGFR), thereby restraining the proliferation and metastasis of ESCC cells and enhancing apoptosis (21). In this study, we demonstrated that KCNQ1OT1 could function as a ceRNA to upregulate *EGFR* expression by competing for miR-133b, thus exerting an oncogenic effect in ESCC.

## MATERIALS AND METHODS

### Specimen collection

Overall, 51 patients with ESCC who underwent surgery in the hospital between October 2016 and May 2019 were enrolled. All patients were histologically confirmed to have ESCC by pathologists at the Binzhou Medical University Hospital and were not suffering from any other malignancies; no neoadjuvant therapy was administered prior to surgery. The patients were categorized according to the TNM staging system (7^th^ edition) of the American Joint Committee on Cancer. Among the patients, 32 were men and 19 were women (age, 43-71 years; average: 66.3 years). Patients receiving neoadjuvant therapy, patients with esophageal adenocarcinoma, patients with other malignancies, or those with severe cardiovascular diseases were excluded. Cancerous and paracancerous tissues (>5 cm from the margin) were collected from the enrolled patients during surgery and were rapidly preserved in liquid nitrogen at -196°C. Informed consent was obtained from each subject, and the study was approved by the Ethics Committee of the Binzhou Medical University Hospital (Ethical approval number: 2016-05.).

### Cell culture and transfection

Human ESCC cell lines (KYSE150, KYSE30, KYSE450, EC9706, and EC109 cells) and human esophageal epithelial cells (Het-1A) were obtained from the American Type Culture Collection (ATCC, Rockville, MD, USA). The cells were cultured in Dulbecco’s modified Eagle’s medium (DMEM, Thermo Fisher Scientific, Shanghai, China) containing 10% fetal bovine serum (FBS, Sciencell Research, Carlsbad, CA, USA) at 37°C in 5% CO_2_.

pcDNA empty vector (normal control, NC), pcDNA-KCNQ1OT1 (KCNQ1OT1), scrambled siRNA (normal control, si-NC), small interfering RNAs against KCNQ1OT1 (si-KCNQ1OT1#1 and si-KCNQ1OT1#2), miRNA control (mimics NC), miR-133b mimics, an inhibitor control (inhibitor NC), and miR-133b inhibitors were obtained from GenePharma Co., Ltd. (Shanghai, China). The KYSE30 and KYSE150 cells were transfected using Lipofectamine^TM^ 2000 (Invitrogen, Carlsbad, CA, USA). Quantitative real-time polymerase chain reaction (qRT-PCR) was used to measure the transfection efficiency after 24h of transfection.

### qRT-PCR

Total RNA was extracted from the tissues and cell lines using TRIzol reagent (Invitrogen, Carlsbad, CA, USA). One Step PrimeScript miRNA cDNA synthesis kit (Takara, Dalian, China) and PrimeScript RT kit (Takara, Dalian, China) were used to reverse transcribe the miRNA and mRNA into cDNA, respectively; qRT-PCR was then performed using SYBR Green PCR reagent (Takara, Dalian, China) on an ABI PRISM 7900HT Sequence Detection System (Applied Biosystems Inc., Foster City, CA, USA). *U6* was used as the internal reference for miR-133b, and *GAPDH* was used as the internal reference for KCNQ1OT1 and *EGFR*. The relative mRNA expression levels of KCNQ1OT1, miR-133b, and *EGFR* were calculated using the 2^-ΔΔCt^ method. The primer sequences used are listed in Table 1 (22).

### Cell counting kit-8 (CCK-8) analysis

After the cells in each group grew to the logarithmic growth phase, they were trypsinized and inoculated into 96-well plates (2×10^3^ cells/well). Subsequently, the cells were cultured for 24, 48, and 72h. At each time point, 10 μL of CCK-8 solution (Dojindo, Kumamoto, Japan) was added to each well, followed by incubation for 2h. Next, the absorbance of the samples in each well was measured at 450 nm using a microplate reader.

### EdU assay

Briefly, KYSE30 and KYSE150 cells from the logarithmic growth phase were inoculated into 96-well plates (2×10^3^ cells/well). After 12h, the cells were incubated with EdU medium (Beyotime Biotechnology, Shanghai, China) at a final concentration of 10 μmol/L for 24h. The medium was discarded, the cells were rinsed with PBS, and fixed with paraformaldehyde for 30 min. According to the manufacturer’s instructions, Apollo staining solution was added, followed by incubation for 30 min and staining with Hoechst staining solution for 30 min. The cells were then rinsed with PBS, and three fields of view were randomly photographed under a fluorescence microscope. The EdU positivity was calculated as follows: EdU-positive cell labeling rate (%) = [green fluorescent (EdU-positive) cells/blue fluorescent (Hoechst-positive) cells] × 100%.

### Flow cytometry analysis

An Annexin V-FITC/propidium iodide (PI) double staining kit (Yeasen Biotech Co., Ltd, Shanghai, China) was used to detect ESCC cell apoptosis. Briefly, after the medium was discarded, the cells were rinsed twice with pre-chilled PBS and resuspended in 1× binding buffer. Following the addition of 5 μL of Annexin V-FITC staining solution and 5 μL of PI solution, the cell suspension was mixed thoroughly and incubated for 15 min in the dark; the apoptotic cells were determined using a flow cytometer (BD Biosciences, San Jose, CA, USA) within 1h.

### Transwell experiment

For the invasion assay, Transwell chambers (Biosharp, Hefei, China) were precoated with Matrigel (30 μg/well; BD, San Jose, CA, USA). The transfected ESCC cells (2×10^4^ cells/well) suspended in serum-free medium were seeded into the upper chamber, and the lower chamber was filled with complete medium. After continuous culture for 48h, the cells remaining on the upper surface of the membrane were wiped off with a cotton swab, and the ESCC cells on the lower membrane surface were fixed with formalin and stained with crystal violet solution. Five visual fields were selected randomly from each group to count the stained cells, and the average was calculated. In the migration assay, except for the addition of Matrigel to the Transwell membrane, all other procedures were the same as those in the invasion experiment.

### Cytoplasmic and nuclear RNA fractionation

Nuclear and cytoplasmic extraction reagents (Thermo Fisher, Carlsbad, CA, USA) were used for the separation of the nuclear and cytoplasmic fractions, respectively, and qRT-PCR was used to determine the expression of KCNQ1OT1 in the nucleus and cytoplasm of ESCC cells, respectively. *GAPDH* and *U6* were used as positive controls for cytoplasmic and nuclear RNA, respectively.

### Western blotting

The total protein of ESCC cells was extracted with RIPA buffer (Beyotime, Shanghai, China) on ice, and the concentration of the protein samples in each group was determined using a BCA kit (Beyotime, Shanghai, China). Equal amounts of proteins were taken from each group and separated by 12% SDS-PAGE before transferring the proteins to PVDF membranes (Millipore, Billerica, MA, USA). The membranes were blocked with 5% skim milk for 2h at room temperature. Primary antibodies against *EGFR*, *PI3K*, *AKT*, E-cadherin, N-cadherin, *MMP-2*, *MMP-9*, and *GAPDH* were added, followed by incubation overnight at 4°C. On the second day, the primary antibodies were discarded. After washing the PVDF membrane with TBST buffer, the membranes were incubated with secondary antibodies for 1h at room temperature. Protein bands were detected using an enhanced chemiluminescence (ECL) system (Biossci, Wuhan, China). The antibodies used in this study, including anti-*EGFR* (ab52894, 1:1000), anti-*PI3K* (ab32089, 1:500), anti-*AKT* (ab179463, 1:500), anti-E-cadherin (ab194982, 1:1000), anti-N-cadherin (ab18203, 1:500), anti-*MMP-2* (ab97779, 1:1000), anti-*MMP-9* (ab219372, 1:1000), and anti-GAPDH antibodies (ab181602, 1:2000), were procured from Abcam (Cambridge, UK).

### Dual-luciferase reporter gene experiment

First, the binding sites on the KCNQ1OT1 sequence and the 3′-UTR of *EGFR* for miR-133b were predicted by bioinformatics analysis, and the fragments containing the binding sites were amplified. The amplified fragments were then inserted into the luciferase reporter vector (Ambion, Austin, TX, USA) to construct the KCNQ1OT1 wild-type reporter (KCNQ1OT1-WT) and the *EGFR* wild-type reporter (*EGFR*-WT). The KCNQ1OT1 mutant reporter (KCNQ1OT1-MUT) and the *EGFR* mutant reporter (*EGFR*-MUT) were constructed by mutating partial nucleotides in the binding site using site-directed mutagenesis. HEK-293T cells were co-transfected with the above-mentioned plasmids and miR-133b mimics (or mimics NC), and ultimately, the luciferase activity of each group was detected using a dual-luciferase reporter gene detection kit (Promega, Madison, WI, USA) according to the manufacturer’s instructions.

### RNA immunoprecipitation (RIP) assay

The interaction between KCNQ1OT1 and miR-133b in ESCC cells was analyzed using the EZ-Magna RNA-binding protein immunoprecipitation kit (Millipore, Billerica, MA, USA). Cells were lysed and the lysate was mixed with magnetic beads coupled with anti-Ago2 antibody or IgG (Millipore, Billerica, MA, USA) in RIP buffer. After incubation at 4°C for 8h, the immunoprecipitated RNA was extracted using the TRIzol method and then reverse transcribed to cDNA. Finally, the expression of KCNQ1OT1 and miR-133b in the immunoprecipitate was analyzed by qRT-PCR.

### Statistical analysis

Statistical software SPSS (version 20.0; SPSS, Inc., Chicago, IL, USA) and GraphPad Prism 8.0 (GraphPad Software, Inc., La Jolla, CA, USA) were used for statistical analysis. All experiments were repeated at least three times. The experimental data were expressed as the mean ± standard deviation (SD). The Shapiro-Wilk normality test was performed to analyze the distribution normality of the gene expression data of the tissues. Student’s *t*-test was used for comparison between two groups, one-way ANOVA with Bonferroni *post hoc* test and two-way ANOVA with Bonferroni *post hoc* test were applied for comparisons among multiple groups. Pearson’s correlation test was used to investigate correlations among the expression levels of KCNQ1OT1, miR-133b, and *EGFR* mRNA. *p*<0.05 indicated statistical significance.

## RESULTS

### KCNQ1OT1 and EGFR were highly expressed whereas miR-133b showed low expression in ESCC tissues and cells

**Figure 1:**
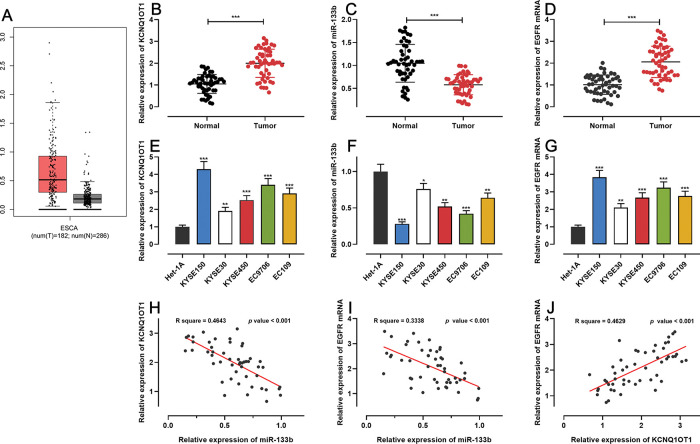
The expression of KCNQ1OT1, miR-133b, and *EGFR* in ESCC. **A.** The expression of KCNQ1OT1 in ESCC samples was analyzed using the online database GEPIA. **B-D.** The mRNA expression levels of KCNQ1OT1, miR-133b, and *EGFR* in 51 pairs of ESCC tissues and non-tumor tissues were determined by qRT-PCR, and paired *t*-tests were used for comparison. **E-G.** The mRNA expression levels of KCNQ1OT1, miR-133b, and *EGFR* in ESCC cells were determined by qRT-PCR, and one-way analysis of variance was used for comparison. **H-J.** Pearson’s correlation analysis was performed to assess the associations among KCNQ1OT1, miR-133b, and *EGFR* mRNA expression levels in ESCC tissues, and Pearson’s correlation test was used to investigate these correlations. *p<0.05, **p<0.01, and ***p<0.001. qRT-PCR, quantitative real-time polymerase chain reaction (qRT-PCR).

First, we analyzed KCNQ1OT1 expression in ESCC using the GEPIA database (http://gepia.cancer-pku.cn/), and found that KCNQ1OT1 expression was remarkably upregulated in ESCC tissues relative to that in normal tissues (Figure 1A). Next, qRT-PCR was performed to detect KCN-Q1OT1, miR-133b, and *EGFR* mRNA expression in 51 pairs of patient specimens. The results suggested that the mRNA expression levels of both KCNQ1OT1 and *EGFR* were increased, whereas miR-133b expression was decreased in ESCC tissues (Figure 1B-D). Similarly, the KCNQ1OT1 and *EGFR* mRNA expression levels in ESCC cell lines were markedly higher than those in normal human esophageal epithelial cells, whereas the miR-133b expression was significantly lower (Figure 1E-G). Further, the median expression levels of KCNQ1OT1 in 51 ESCC tissue samples was used to classify the high expression group (26 cases) and low expression group (25 cases). Upregulation of KCNQ1OT1 expression in ESCC tissues was associated with larger tumor size and advanced TNM stage (Table 2). Pearson’s correlation analysis revealed a positive relationship between the KCNQ1OT1 and *EGFR* mRNA expression levels, a negative correlation between the KCNQ1OT1 and miR-133b expression levels, and a negative correlation between the *EGFR* mRNA and miR-133b expression levels in ESCC tissues (Figure 1H-J).

### KCNQ1OT1 enhanced the proliferation and restrained the apoptosis of ESCC cells *in vitro*

**Figure 2:**
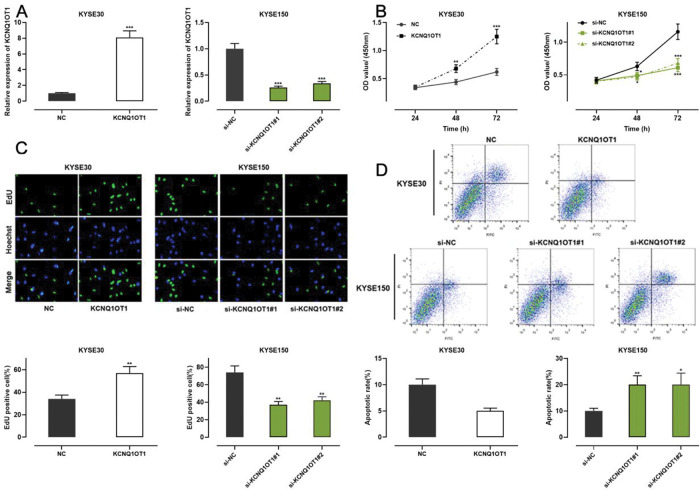
The role of KCNQ1OT1 in ESCC cell proliferation and apoptosis. **A.** KCNQ1OT1 overexpression plasmid and siRNA targeting KCNQ1OT1 were transfected into ESCC cells. qRT-PCR was used to determine the transfection efficiency, which was compared using Student’s *t*-test. **B, C.** The effects of KCNQ1OT1 on ESCC cell proliferation were detected using the CCK-8 method and EdU experiments, and two-way ANOVA and Student’s *t-*test were used for the comparisons, respectively. **D.** Flow cytometry analysis was used to detect the effects of KCNQ1OT1 on apoptosis, and Student’s *t-*test was used for comparisons. **p<0.01 and ***p<0.001. NC, normal control; si-NC, normal control siRNA.

Among all ESCC cell lines analyzed in this study, KCNQ1OT1 expression was the lowest in KYSE30 cells and the highest in KYSE150 cells (Figure 1E). Therefore, we performed gain-of-function experiments in KYSE30 cells and loss-of-function experiments in KYSE150 cells. A CCK-8 assay, EdU assay, and apoptosis analysis were conducted to investigate the functional role of KCNQ1OT1 in these cells. pcDNA KCNQ1OT1, KCNQ1OT1 siRNA (si-KCNQ1OT1#1 and si-KCNQ1OT1#2), pcDNA normal control (NC), and negative control (si-NC) were constructed for these above experiments (Figure 2A). The CCK-8 and EdU assays demonstrated that KCNQ1OT1 overexpression facilitated KYSE30 cell proliferation, compared to the case for cells in the NC group, whereas KCNQ1OT1 knockdown exerted the opposite effects on KYSE150 cells (Figure 2B, C). Flow cytometry revealed that KCNQ1OT1 overexpression repressed apoptosis in KYSE30 cells, whereas KCNQ1OT1 knockdown induced apoptosis in KYSE150 cells (Figure 2D).

### KCNQ1OT1 accelerated the migration, invasion, and epithelial-mesenchymal transition (EMT) of ESCC cells

**Figure 3:**
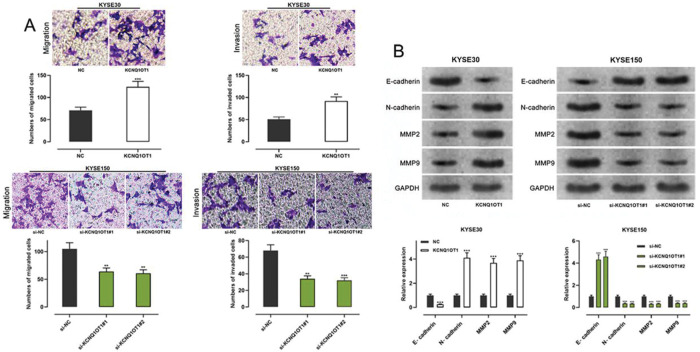
The impact of KCNQ1OT1 on migration, invasion, and EMT processes on ESCC cells. **A.** Transwell experiments were employed to detect the effects of KCNQ1OT1 on ESCC cell migration. **B.** Western blotting was used to detect the effects of KCNQ1OT1 on the protein expression of E-cadherin, N-cadherin, MMP-2, and MMP-9. Student’s *t-*test was used for comparing the results. **p<0.01 and ***p<0.001. NC, normal control; si-NC, siRNA normal control; EMT, epithelial-mesenchymal transition.

Transwell experiments were performed to determine the effect of KCNQ1OT1 on cell migration and invasion. The results indicated that KCNQ1OT1 overexpression enhanced the migration and invasion abilities of KYSE30 cells, whereas KCNQ1OT1 knockdown suppressed the migration and invasion abilities of KYSE150 cells (Figure 3A). EMT, which is correlated with the loss of epithelial characteristics and gain of mesenchymal characteristics, confers a high invasive potential to cancer cells. The results of the western blot analysis indicated that KCNQ1OT1 overexpression remarkably downregulated E-cadherin expression and upregulated N-cadherin, MMP-2, and MMP-9 expression, whereas the knockdown of KCNQ1OT1 had the opposite effects (Figure 3B). These results suggest that KCNQ1OT1 promotes EMT and aggressiveness in ESCC cells.

### KCNQ1OT1 targeted miR-133b as a miRNA sponge

**Figure 4:**
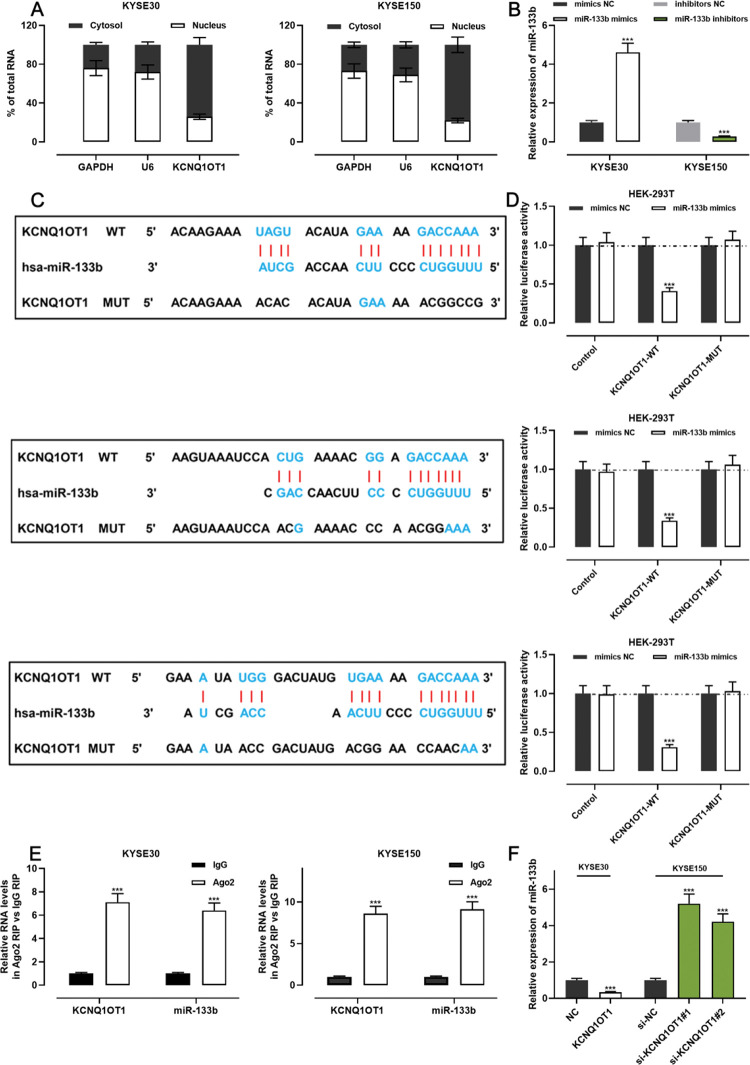
KCNQ1OT1 specifically targets miR-133b. **A.** The localization of KCNQ1OT1 in KYSE30 and KYSE150 cells was analyzed by qRT-PCR. and Student’s *t*-test was used for comparison. **B.** The transfection efficiency of miR-133b mimics or miR-133b inhibitors in KYSE30 and KYSE150 cells was determined by qRT-PCR and was compared using the Student’s *t*-test. **C.** The online database LncBase Predicted v.2 was used to predict the binding site between KCNQ1OT1 and miR-133b. **D.** KCNQ1OT1-WT or KCNQ1OT1- MUT luciferase reporter was co-transfected with miR-133b mimics into HEK-293T cells; the luciferase activity in each group was measured, and the luciferase activities were compared using Student’s *t*-test. **E.** Direct interaction between KCNQ1OT1 and miR-133b was analyzed using RIP experiments; Student’s *t*-test was used for comparison. **F.** qRT-PCR was used to detect the effects of KCNQ1OT1 on miR-133b expression in ESCC cells, and the results were compared using Student’s *t-*test. ***p<0.001. WT, wild-type; MUT, mutant.

The qRT-PCR analysis indicated that KCNQ1OT1 was enriched in the cytoplasm but not in the nucleus, suggesting that it exerts its biological functions via the “ceRNA” mechanism (Figure 4A). We transfected miR-133b mimics into KYSE30 cells and transfected miR-133b inhibitors into KYSE150 cells; the qRT-PCR analysis then showed that the transfection was successful (Figure 4B). To investigate the mechanisms underlying the action of KCNQ1OT1, the LncBase Predicted database (carolina.imis.athena-innovation.gr/diana_tools/web/index.php) was employed to analyze the target recognition sequence of KCNQ1OT1 for miRNAs, and the result revealed that miR-133b had a sequence complementary to that of KCNQ1OT1 (Figure 4C). We then performed a dual-luciferase reporter assay to verify this binding relationship, and the data revealed that miR-133b mimics could inhibit the luciferase activity of the KCNQ1OT1-WT reporter, whereas it had no notable effect on the luciferase activity of the KCNQ1OT1-MUT reporter (Figure 4D). To further verify whether KCNQ1OT1 interacted with miR-133b, a RIP assay was performed on the KYSE30 and KYSE150 cell extracts using antibodies against Ago2, and the results implied that KCNQ1OT1 and miR-133b were significantly enriched in the immunoprecipitate containing Ago2 (Figure 4E). Further, KCNQ1OT1 overexpression restrained the miR-133b expression in KYSE30 cells, whereas KCNQ1OT1 knockdown enhanced miR-133b expression in KYSE150 cells (Figure 4F). These results indicate that KCNQ1OT1 directly targets miR-133b and negatively modulates the expression of the latter.

### KCNQ1OT1 enhanced the proliferation and metastasis of ESCC cells by adsorbing miR-133b and impeded their apoptosis

**Figure 5:**
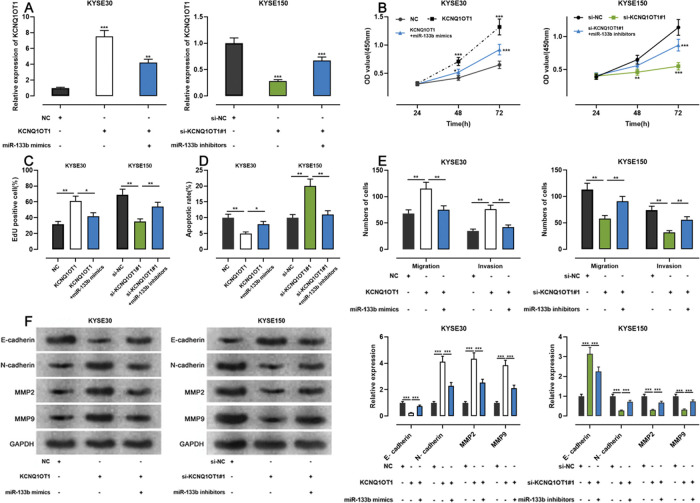
Effects of KCNQ1OT1 on ESCC cell proliferation, apoptosis, metastasis, and EMT. **A.** KCNQ1OT1 overexpression plasmid was co-transfected with the miR-133b mimic si-KCNQ1OT1#1 or miR-133b inhibitor into KYSE30 and KYSE150 cells, respectively. qRT-PCR was used to detect the transfection efficiency; Student’s *t-*test was used for comparison. **B.** The CCK-8 method was used to detect the effects of KCNQ1OT1 and miR-133b on the proliferation of KYSE30 and KYSE150 cells; the results were compared using two-way ANOVA. **C.** EdU assays were performed to detect the effects of KCNQ1OT1 and miR-133b on cell proliferation, and the results were compared using Student’s *t-*test. **D.** The effects of KCNQ1OT1 and miR-133b on ESCC cell apoptosis were determined by flow cytometry and compared using Student’s *t-*test. **E.** Transwell experiments were conducted to examine the effects of KCNQ1OT1 and miR-133b on the migration and invasion of ESCC cells; Student’s *t-*test was used for the comparisons. **F.** Western blotting was performed to detect the effects of KCNQ1OT1 and miR-133b on the protein expression levels of E-cadherin, N-cadherin, MMP-2, and MMP-9; Student’s *t-*test was used for the comparisons. *p<0.05, **p<0.01, and ***p<0.001. NC, normal control; si-NC, siRNA normal control; EMT, epithelial-mesenchymal transition.

To examine whether KCNQ1OT1 promoted the growth and metastasis of ESCC cells through the adsorption of miR-133b, we co-transfected KYSE30 cells with a KCNQ1OT1 overexpression plasmid and miR-133b mimics, and co-transfected KYSE150 with siRNA targeting KCNQ1OT1 and miR-133b inhibitors. Next, qRT-PCR was used to verify whether the transfection was successful (Figure 5A). Subsequently, we used the CCK-8, EdU, flow cytometry, transwell, and western blotting assays to detect changes in the proliferation, apoptosis, migration, invasion, and expression levels of EMT-related proteins in the cells, respectively. The results showed that KCNQ1OT1 overexpression enhanced cell proliferation, migration, and invasion; restrained apoptosis; upregulated N-cadherin, MMP-2, and MMP-9 expression; and downregulated E-cadherin expression. In contrast, co-transfection with the miR-133b mimics abolished these effects. Conversely, KCNQ1OT1 knockdown exerted contrasting effects, which were partially reversed by miR-133b inhibitors (Figure 5B-F). These findings imply that KCNQ1OT1 promotes ESCC cell proliferation and metastasis and suppresses their apoptosis by regulating miR-133b expression.

### KCNQ1OT1/miR-133b/EGFR axis regulated ESCC progression via the PI3K/AKT signaling pathway

**Figure 6:**
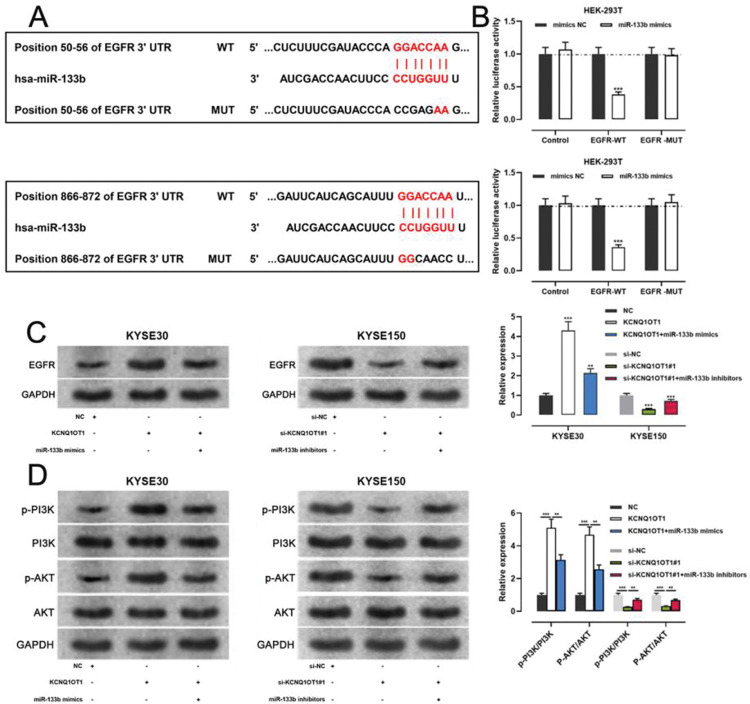
KCNQ1OT1 upregulates *EGFR* expression by adsorbing miR-133b. **A.** The TargetScan database was used to predict the binding sites of miR-133b on the 3′-UTR of *EGFR*. **B.** A dual-luciferase reporter assay system was employed to detect the binding relationship between *EGFR* and miR-133b; Student’s *t*-test was used for comparing the results. **C.** The effects of KCNQ1OT1 and miR-133b on *EGFR* expression were detected by qRT-PCR and western blotting, and the results were compared using Student’s *t-*test. **D.** Western blotting was used to detect the expression of PI3K/AKT signaling pathway-related proteins; Student’s *t-*test was used for comparing the results. **p<0.01 and ***p<0.001. WT, wild-type; MUT, mutant; 3′ UTR, 3′ untranslated region.

miR-133b has been reported to target and regulate *EGFR*(20). To verify this targeting relationship, we identified a potential binding site for miR-133b in the 3′-UTR of *EGFR* using the TargetScan database (Figure 6A). We found that miR-133b mimics suppressed the luciferase activity of the *EGFR*-WT reporter the a dual-luciferase reporter gene assay, but exerted no notable effects on luciferase activity in the *EGFR*-MUT group (Figure 6B). Moreover, western blotting showed that KCNQ1OT1 overexpression facilitated *EGFR* expression, which was counteracted by miR-133b mimics; KCNQ1OT1 depletion inhibited *EGFR* expression, which was abrogated by miR-133b inhibitors (Figure 6C). To further verify whether KCNQ1OT1 modulated the activity of the PI3K/AKT signaling pathway, we tested the protein expression of p-PI3K, p-AKT, PI3K, and AKT by western blotting. The data implied that KCNQ1OT1 overexpression facilitated the upregulation of p-PI3K and p-AKT expression, which was reversed by miR-133b mimics. Conversely, KCNQ1OT1 knockdown inhibited the expression of p-*PI3K* and p-*AKT*, which was abolished by miR-133b inhibitors (Figure 6D). These data indicated that KCNQ1OT1 activated the PI3K/AKT signaling pathway probably by regulating the miR-133b/*EGFR* molecular axis.

## DISCUSSION

LncRNAs were previously regarded as “junk transcripts.” However, in recent years, lncRNAs have been shown to participate in gene regulation and serve as essential regulators of cell proliferation, cell cycle, differentiation, apoptosis, and migration, exerting tumor-promoting or tumor-suppressive effects (23,24). KCNQ1OT1 is transcribed by RNA polymerase II in an antisense direction from a highly conserved and differentially methylated region (KCNQ1ICR, KvDMR, or IC2) in intron 10 of the KCNQ1 gene, and KCNQ1OT1 interacts with chromatin to regulate the transcription of several genes in an epigenetic manner, playing important roles in the tumorigenesis and development of various cancers (25,26). KCNQ1OT1 is highly expressed in colorectal cancer (CRC), and its high expression predicts an unfavorable prognosis; functionally, KCNQ1OT1 facilitates CRC cell growth and metastasis by modulating the miR-217/ZEB1 molecular axis (27). KCNQ1OT1 expression is also upregulated in osteosarcoma and non-small cell lung cancer tissues, and its high expression indicates the adverse prognosis of patients with these diseases (28,29). Moreover, survival analysis demonstrated that the survival time of ESCC patients with KCNQ1OT1 overexpression is remarkably shorter than that of patients with low KCNQ1OT1 expression (18). In this study, we found that KCNQ1OT1 expression was upregulated in ESCC specimens, and its high expression was associated with adverse pathological parameters in ESCC patients. Further, KCNQ1OT1 enhances the proliferation, migration, invasion, and EMT of cancer cells, while repressing their apoptosis. These results suggest that KCNQ1OT1 is a promising marker and therapeutic target for ESCC.

Previous studies have shown that miR-133b is abnormally expressed in diverse malignancies, including ESCC (30-35). MiR-133b impedes CRC cell proliferation and blocks the cell cycle by targeting *NUP214*, and miR-133b expression is negatively regulated by LINC00114 (34). MiR-133b restrains ESCC growth and metastasis by suppressing the FSCN1/β-catenin signaling pathway, and lncRNA TTN-AS1 directly targets miR-133b and negatively regulates its expression (35). In this study, we found that KCNQ1OT1 is a ceRNA that reduces the availability of miR-133b. Further experiments confirmed that KCNQ1OT1 accelerates the growth and metastasis of ESCC by adsorbing miR-133b.

*EGFR* is a 170-kDa transmembrane protein that exhibits tyrosine kinase activity following binding to its ligands (36). *EGFR* belongs to the ErbB oncogene family and is widely expressed in epithelial cells. It also exerts a vital effect on tumorigenesis by interacting with epidermal growth factor (EGF) (37). Excessive activation of EGFR activates multiple downstream signaling pathways, such as the MEK/ERK/MAPK, PI3K/AKT/mTOR, and JAK1/STAT3/5 signaling pathways), thereby enhancing the tumorigenesis, progression, and metastasis of various cancers (38,39). Reportedly, mediator complex subunit 19 (Med19) interacts with EGFR and increases its expression; it activates the EGFR/MEK/ERK pathway and exerts its oncogenic activity in an EGFR-dependent manner in breast cancer (40). In liver cancer, EGFR activates hepatocyte growth factor (HGF) expression by repressing miR-26a/b expression, and the upregulation of paracrine HGF, which binds to the c-MET receptor of migrated cancer cells, promotes the proliferation of metastatic cancer cells (41). In ESCC, EGFR increases the phosphorylation levels of ERK, AKT, and PI3K, and enhances ESCC cell proliferation, metastasis, and EMT by activating the PI3K/AKT and MAPK/ERK signaling pathways; *EGFR* expression is negatively regulated by miR-133b (20). *EGFR* is considered an important therapeutic target for multiple cancers, but the mechanism underlying its dysregulation remains unclear. In this study, our data revealed that KCNQ1OT1 could upregulate EGFR expression by adsorbing miR-133b. Additionally, KCNQ1OT1 activated PI3K/AKT signaling, which is a pathway present downstream of EGFR, to participate in ESCC progression.

In summary, our results reveal that KCNQ1OT1 is abnormally overexpressed in ESCC, and that its high expression implies an adverse prognosis. Functionally and mechanistically, KCNQ1OT1 functions as an oncogenic lncRNA in the growth and metastasis of ESCC cells by activating the EGFR/PI3K/AKT signaling pathway via the adsorption of miR-133b. Thus, our study helps clarify the mechanism underlying ESCC progression and provides clues for the diagnosis and treatment of ESCC.

## AUTHOR CONTRIBUTIONS

Zhang Q and Zhang L conceived the study, provided advice, and prepared the manuscript. Xu H and Miao J conducted the experiments and wrote the manuscript. Liu S and Liu H participated in data collection and analysis. All authors have reviewed and approved the final version of the manuscript.

## Figures and Tables

**Table 1:** Primer sequences used for qRT-PCR.

KCNQ1OT1	F: ACTCACTCACTCACTCACT R: CTGGCTCCTTCTATCACATT
miR-133b	RT: GTCGTATCCAGTGCAGGGTCCGAGGTATTCGC ACTGGATACGACTAGCTG
	R: CCGTTTGGTCCCCTTCAAC
*EGFR*	F: GGTCTT GAAGGCTGTCCAACG R: CCTCAAGAGAGCTTGGTT GGG
*U6*	F: CTGCTTCGGCAGCACA R: AACGCTTCACGAATTTGCGT
*GAPDH*	F: TCTCTGCTCCTCCTGTTC R: GTTGACTCCGACCTTCAC

Abbreviations: F, forward; R, reverse; RT, reverse transcription.

**Table 2:** Correlations between KCNQ1OT1 expression and clinicopathological parameters of ESCC patients.

Pathological Parameters	N (%)	KCNQ1OT1 expression		χ2	*p-*value
		High	Low		
N (%)	51 (100%)	26 (51%)	25 (49%)		
Gender				0.9544	0.3286
Male	32 (62%)	18	14		
Female	19 (38%)	8	11		
Age (years)				0.6918	0.4056
<60	15 (29%)	9	6		
≥60	36 (71%)	17	19		
Tumor size (cm)				5.9928	0.0144*
<5	18 (35%)	5	13		
≥5	33 (65%)	21	12		
TNM stage				4.2384	0.0395*
I+II	12 (24%)	3	9		
III+IV	39 (76%)	23	16		
Histological grade				0.4727	0.4917
Well	22 (43%)	10	12		
Poor	29 (57%)	16	13		
Lymph node metastasis				4.4806	0.4882
Negative	24 (47%)	11	13		
Positive	27 (53%)	15	12		

*p<0.05.
